# Corneal and anterior chamber morphology in patients with nonınfectious ıntraocular ınflammation

**DOI:** 10.5935/0004-2749.20210030

**Published:** 2021

**Authors:** Ebru Nevin Cetin, Kerem Bozkurt, Selen Akbulut, Gökhan Pekel, Murat Taşcı, Veli Çobankara

**Affiliations:** 1 Department of Ophthalmology, Pamukkale University, Denizli, Turkey; 2 Department of Ophthalmology, Denizli State Hospital, Denizli, Turkey; 3 Department of Rheumatology, Pamukkale University, Denizli, Turkey

**Keywords:** Anterior chamber, Inflammation, Endothelium, corneal, Cell count, Uveitis, Câmara anterior, Inflamação, Epitélio posterior, Contagem de células, Uveites

## Abstract

**Purpose:**

To evaluate the corneal and anterior chamber morphology in phakic eyes with noninfectious intraocular inflammation.

**Methods:**

This study included 59 eyes with active uveitis, 62 with inactive uveitis, and 95 healthy eyes. Corneal endothelial cell density, hexagonal cell ratio, coefficient of variation (CV), corneal thickness and volume, maximum keratometry, and anterior chamber volume and depth (ACD) measurements were performed using a specular microscope and Pentacam HR.

**Results:**

The mean duration of uveitis was 24.6 ± 40.5 (0-180) months. The mean number of uveitis attacks was 2.8 ± 3.0 (1-20). Coefficient of variation was significantly higher in the active uveitis group compared with inactive uveitis group (p=0.017, Post Hoc Tukey). Anterior segment parameters other than coefficient of variation were not significantly different between active/inactive uveitis and control groups (p>0.05). Multiple linear regression analysis showed that coefficient of variation was greater in active uveitis compared with inactive uveitis after adjusting for the duration of uveitis, type of uveitis, having a rheumatologic disease, and having immunosuppressive treatment (p=0.003). The duration of uveitis and number of attacks were not significantly correlated with ocular parameters (p>0.05, Spearman’s correlation). The difference in parameters was not significant based on uveitis type (p>0.05).

**Conclusions:**

Coefficient of variation was higher in eyes with active uveitis than that in eyes with inactive uveitis, whereas corneal endothelial cell density and anterior chamber morphology did not significantly differ between active/inactive uveitis and control groups.

## Abstract (pt)

**Objetivo:**

Avaliar a morfologia da córnea e da câmara anterior em olhos fácicos com inflamação intraocular não infecciosa.

**Métodos:**

Esse estudo incluiu 59 olhos com uveíte ativa, 62 olhos com uveíte inativa e 95 olhos saudáveis. A densidade de células endoteliais da córnea, a proporção de células hexagonais, o coeficiente de variação, o volume e a espessura da córnea, a ceratometria máxima e o volume e profundidade da câmara anterior foram medidos com um microscópio especular e uma Pentacam HR.

**Resultados:**

A duração média da uveíte foi de 24,6 ± 40,5 (0-180) meses. O número médio de crises de uveíte foi de 2,8 ± 3,0 (1-20). O coeficiente de variação foi significativamente maior no grupo com uveíte ativa do que no grupo com uveíte inativa (p=0,017, Tukey *post-hoc*). Não houve diferença significativa nos demais parâmetros do segmento anterior entre os grupos com uveíte ativa, com uveíte inativa e controle (p>0,05). A análise de regressão linear múltipla demonstrou que o coeficiente de variação foi maior na uveíte ativa do que na uveíte inativa, após ajustes para a duração e tipo de uveíte e a presença ou não de doença reumática e de tratamento imunossupressor (p=0,003). A duração da uveíte e o número de crises não demonstraram correlação significativa com os parâmetros oculares (p>0,05, correlação de Spearman). A diferença nos parâmetros não demonstrou correlação significativa com o tipo de uveíte (p>0,05).

**Conclusões:**

O coeficiente de variação foi maior nos olhos com uveíte ativa do que naqueles com uveíte inativa, ao passo que a densidade de células endoteliais e a morfologia da câmara anterior não mostraram diferenças significativas entre os grupos com uveíte ativa, com uveíte inativa e controle.

## INTRODUCTION

The healthy corneal endothelium is crucial to maintain the optical transparency of the cornea. The corneal endothelium requires lack of proliferation, and its loss is mainly accomplished by enlargement and spread of neighboring cells to cover any defective area, resulting in increased cellular pleomorphism, and decreased number of hexagonal cells^(1)^. Therefore, the variation in cell size, endothelial cell density (ECD), and number of hexagonal cells are parameters of healthy corneal endothelium. Aging, trauma, drugs, contact lens, glaucoma, diabetes, and intraocular surgery are several factors that influenced corneal endothelium^(2)^.

Chronic and severe intraocular inflammation are often associated with ocular complications, such as glaucoma or cataract^(3,4)^. Therefore, patients with uveitic eyes are highly at risk of requiring intraocular surgery. In case of corneal endothelial damage, intraocular surgery might be even more challenging in uveitic eyes combined with posterior synechia, small pupil, and fragile zonules^(3)^. In previous studies, anterior segment inflammation was associated with lower ECD and variation of morphologic features including cell size and hexagonal cell ratio^(5-8)^. Another study showed that lower mean corneal hysteresis and corneal resistance factor found in anterior uveitis^(9)^. Some investigators also reported corneal thickness (CT) alterations that tended to increase during an attack and decrease with the treatment for anterior uveitis^(10-13)^. Laboratory studies suggested that intraocular inflammatory reactions mediated by inflammatory cytokines in the aqueous humor might be responsible for corneal alterations in uveitic eyes^(14-16)^.

Although previous studies revealed some alterations in the corneal endothelium and CT, small sample sizes, lack of appropriate control groups, or discrimination of active/inactive uveitis cause difficulties in making clear comments on the effects of intraocular inflammation on corneal structures^(5-7)^. This study aimed to assess the corneal ECD and morphological features as well as other anterior segment parameters including corneal volume and thickness, maximum keratometry, anterior chamber depth (ACD), and anterior chamber volume (ACV) as mea sured by Pentacam HR in patients with noninfectious intraocular inflammation. To our knowledge, this is the largest study evaluating the effects of active/previous anterior segment inflammation on corneal and anterior chamber morphology in phakic eyes.

## METHODS

This prospective cross-sectional study was conducted at a tertiary setting during the approval of the Institutional Review Board and adhered to the principles of the Declaration of Helsinki. Data at the time of enrollment to the study was compared between the study and control groups. All participants provided written informed consent. The study group consisted of patients with noninfectious intraocular inflammation, whereas the control group included healthy participants. The study was group further divided into two groups based on uveitis activity. All participants underwent a comprehensive ophthalmic examination, including anterior and posterior segment examination and intraocular pressure meas urements. Anterior chamber cells and uveitis category were scored based on definitions published by the Standardization of Uveitis Nomenclature Working Group^(17)^. The time since uveietis diagnosis, the coexistent of systemic inflammatory diseases, number of previous uveitis attacks, and medications (topical/intraocular/systemic steroids, anti-metabolites, and biological agents) were also recorded. Exclusion criteria were having ocular diseases that affect the cornea and anterior segment (corneal pathology, infectious keratitis/conjunctivitis, etc.), history of ocular trauma, intraocular surgery, and contact lens wear; and having poor quality images on specular microscope and Pentacam HR.

A non-contact specular microscope was used (CEM530, Nidek, Japan) for corneal endothelium assessment. The device provides central, paracentral, and peripheral views with graphics and color-coded cell images. Corneal ECD, hexagonal cell ratio, and coefficient of variation (CV) data were used for analysis. CV indicated the standard deviation of cell area per mean cell area.

Pentacam HR (Oculus, Wetzlar, Germany) provides a three-dimensional model of the anterior segment, ele vation maps of the anterior and posterior corneal sur faces, anterior and posterior corneal power calculations, pachymetric and biometric measurements of the anterior segment using a rotating Scheimpflug camera (180°), and monochromatic slit light source combined with a static camera. Only scans with quality specification examination of “OK” were considered for analysis. Central CT and volume, maximum keratometry, ACV, and ACD measurements were used for analysis.

In the control group, measurements of only one eye per patient were considered for analysis as findings of the right and left eyes were almost identical. In patients with bilateral uveitis, both eyes were analyzed since each uveitis attack may have different severities and influence of attack may vary between the eyes.

Statistical analysis was performed by SPSS statistical software (SPSS 11.0.0 for MS Windows; SPSS Inc., Chicago, IL). Kolmogorov-Smirnov test was used to determine the data distribution. Parametric tests were used to compare homogenously distributed data, whereas nonparametric tests were used to compare non-homogenously distributed data. Categorical variables were compared using the χ2-test. A p value <0.05 was considered significant.

## RESULTS

A total of 216 eyes were included in this study. The uveitis group consisted of 121 eyes (59 active and 62 inactive uveitis) from 92 patients, whereas the control group comprised 95 eyes from 95 subjects. The mean age was 41.2 ± 14.5 and 41.3 ± 13.9 in the study and control groups, respectively (p=0.986). Female/male ratio was 51/41 in the uveitis group, but 55/40 in the control group (p=0.734). All participants had phakic eyes. Associated rheumatologic diseases were Behcet’s disease in 36 (39.1%) patients, ankylosing spondylitis in 10 (10.9%), Crohn’s disease in 2 (2.2%), rheumatoid arthritis in 2 (2.2%), psoriasis in 1 (1.1%), Sjogren’s syndrome, and sarcoidosis in 1 (1.1%).

In the study group, the mean uveitis duration and number of attacks were 24.6 ± 40.5 (0-180) months and 2.8 ± 3.0 (1-20), respectively. Sixty-seven (55.4%) eyes had anterior uveitis, 10 (8.3%) eyes had intermediate uveitis, 10 (8.3%) eyes had posterior uveitis, and 34 (28.1%) eyes had panuveitis. Among those in the idiopathic group (not related to any systemic diseases), 31 (54.4%) eyes had anterior uveitis, 7 (12.3%) had intermediate uveitis (pars planitis), 6 (10.5%) had posterior uveitis, and 13 (22.8%) had panuveitis. In general, 55 (45.5%) eyes were treated with topical steroids, whereas 7 (5.8%) eyes were treated with dexamethasone implants. Twenty (21.7%) patients were on systemic steroid treatment. Steroid-sparing immunosuppressive agents used were azathioprine (16 patients, 17.4%), colchicine (4,4.3%), infliximab (3, 3.3%), golimumab (1,1.1%), mycophenolate mofetil (1, 1.1%), salazopyrin (1,1.1%), methotrexate (1,1.1%), hydroxychloroq uine (1,1.1%), and cyclosporine (1,1.1%). Ten patients (10.9%) used more than one immunosuppressive agents (combination treatment).

Table 1 shows the comparison of anterior segment parameters among active, inactive uveitis, and control groups. The difference was significant in only CV among the three groups (p=0.023, one-way analysis of variance). CV was significantly higher in the active uveitis group than that in the inactive uveitis group (p=0.017, post hoc Tukey). Multiple linear regression analysis showed CV to be higher in the active uveitis than that in the inactive uveitis group after adjusting the duration and type of uveitis, having a rheumatologic disease, and having immunosuppressive treatment (p=0.003).

**Table 1:** The comparison of anterior segment parameters between groups

	Active uveitis	Inactive uveitis	Control group	p value
ECD	2599 ± 250	2615 ± 287	2575 ± 294.0	0.663
Hexagonal cell ratio	68.1 ± 6.2	68.8 ± 6.5	67.7 ± 5.4	0.505
Coefficient of variation	32.0 ± 6.5	29.0 ± 4.9	30.6 ± 5.9	0.023^*^
CT	551.4 ± 47.2	550.7 ± 33.2	544.2 ± 36.8	0.439
Corneal volume	60.0 ± 4.4	60.2 ± 3.6	59.7 ± 4.0	0.669
Kmax	44.4 ± 1.6	45.0 ± 1.8	44.5 ± 1.8	0.129
ACD	2.93 ± 0.41	2.80 ± 0.39	2.89 ± 0.36	0.161
ACV	168.3 ± 38.1	153.8 ± 34.1	159.2 ± 37.0	0.094

One-Way ANOVA.

* *p*=0.017 for comparison of CV between active and inactive uveitis groups (Post Hoc Tukey).

ECD= Endothelial cell density; CT= Corneal thickness; Kmax= Maximum keratometry; ACD= Anterior chamber depth; ACV= Anterior chamber volume.

The duration of uveitis and number of attacks were not significantly correlated with ocular parameters (p>0.05, Spearman’s correlation). The difference in parameters was not significant based on the uveitis type (p>0.05).

## DISCUSSION

In this study, CV was found to be higher in the active uveitis group than that in the inactive uveitis group after adjusting the duration and type of uveitis, presence a rheumatologic disease, and presence immunosuppressive treatment. The difference in anterior segment parameters was not significant between the uveitis and control groups. To our knowledge, this is the largest study evaluating the corneal and anterior chamber morphology in phakic uveitic eyes.

The corneal endothelium in uveitic eyes may be prone to damage in several ways. Pro-inflammatory cytokines and chemokines had been detected within the ocular fluids or tissues in uveitic eyes^(14,15)^. They were suggested to be responsible for tissue damage and ocular complications in uveitis^(16)^. In an endotoxin-induced uveitis model, investigators observed that corneal edema and significant corneal thickening in the cornea compared with the control group and showed that corneal structure could be altered during inflammation^(18)^.

Corneal endothelial damage may be clinically significant in uveitis in two ways. First, uveitic eyes often require intraocular surgeries due to secondary ocular complications such as cataract and glaucoma^(3,4)^. As intraocular surgery itself is a trauma for endothelium, any additional endothelial damage may further affect the postoperative corneal transparency and visual outcome. Second, CT alterations may affect intraocular pressure measurements and may serve as a confounding factor during the follow-up of uveitis patients with glaucoma^(19)^. Therefore, the effect of intraocular inflammation corneal parameters should be clearly understood.

Previous studies focusing on specular microscopic findings in patients with uveitis reported ECD alterations in a small series^(5,6)^. In recent years, Alfawaz et al. compared corneal endothelium in 84 eyes with active or previous anterior segment intraocular inflammation with a historical population of normal, age-matched participants as the primary control group^(7)^. The authors reported that ECD was lower in eyes with uveitis than in the control eyes, whereas CT was similar between the two groups. Lack of elimination of pseudophakic eyes and a relatively small number of patients in subgroups cause difficulties comparing findings with other studies. In another recent study, Guclu et al. assessed the effects of previous inflammation on corneal ECD and morphology^(8)^. Investigators found a significant decrease in ECD and hexagonal cell ratio, whereas increased CV in the eyes with inactive uveitis was compared to that in control patients. CT was not significantly different between eyes with inactive uveitis and controls.

In this study, CV was significantly higher in active uveitis than that in inactive uveitis group; however, it was not significantly different between inactive uveitis and controls, suggesting that CV alterations, which might reflect the variation in individual cell area caused by stress during active inflammation, were reversible. ECD and hexagonal cell ratio were similar between active, inactive uveitis, and control groups in this study. Previous literature reported that increased CV might occur without ECD changes in certain stressful conditions^(20)^. Persistence of CV alterations in inactive uveitis may be suggested by Guclu et al., which might be associated with the duration of previous active inflammation; however, further studies are needed for further clarification^(8)^.

CT was shown to increase during an acute uveitis attack^(10-13,21,22)^. However, a significant difference was observed in CT between the groups, irrespective of activity; similar to Alfawaz et al. and Guclu et al.’s studies^(7,8)^. Since some patients were diagnosed and started treatment before the referral in our hospital, our examination might not be at the most initial period of the attack and therefore might not reflect the effect of the maximum inflammation level on the anterior segment. The difference in findings of these studies may be related to different timing of examination as well as differences in sample sizes ranging from 13 eyes to 121 eyes as in our study.

One of the limitations in this study is the lack of laser flare photometry measurements. The second limitation is that the number of attacks was recorded based on the information provided by patients without previous records in our hospital. Third, some patients presented to our clinic undergoing treatment alreadly; therefore, measurements were not performed on the maximum inflammation level in the active uveitis group. Fourth, participants in this study were patients undergoing uveitis treatment and under follow-up; therefore, findings did not reflect the chronic results and untreated intraocular inflammation. Final limitation is that 16% of eyes in our series had intermediate or posterior uveitis that may demonstrate minimal or no cells in the anterior chamber.

In conclusion, we found that CV was significantly higher in eyes with active uveitis compared to eyes with inactive uveitis; however, ECD, hexagonal cell ratio, and anterior chamber parameters were similar between uveitic eyes and controls, regardless of uveitis activity. Our findings suggest that some corneal morphological changes appear with intraocular inflammation in the active phase; however, uveitic eyes during the inactive phase are not likely to show significant and permanent anterior segment alterations.
